# The Effect of Exposure to Cd and Pb in the Form of a Drinking Water or Feed on the Accumulation and Distribution of These Metals in the Organs of Growing Wistar Rats

**DOI:** 10.1007/s12011-015-0414-4

**Published:** 2015-06-27

**Authors:** Anna Winiarska-Mieczan, Małgorzata Kwiecień

**Affiliations:** Department of Bromatology and Food Physiology, University of Life Sciences in Lublin, Akademicka 13, 20-950 Lublin, Poland; Department of Animal Nutrition, University of Life Sciences in Lublin, Akademicka 13, 20-950 Lublin, Poland

**Keywords:** Cadmium, Lead, Organs accumulation, Drinking water or feed exposure, Growing rats

## Abstract

The degree of accumulation and distribution of Cd and Pb in the organs of young animals compared to the amount taken in with water or feed have not been thoroughly investigated yet. The experiment aimed to verify whether the source of toxic metals (feed, drinking water) administered to growing rats orally has an influence on the degree of accumulation of Cd and Pb in the organs (brain, spleen, lungs, heart, liver and kidneys). The rats received Cd and/or Pb respectively in the amount of 7 mg and/or 50 mg per 1 kg of feed or per 1 L of distilled water. The rats’ organs accumulated in total about 0.5 % Cd and about 0.71 % Pb consumed with water and about 0.46 % Cd and about 0.63 % Pb taken in with feed. More than 60 % of Cd and more than 70 % of Pb absorbed by the studied organs was accumulated in the liver, and more than 30 % of Cd and 26–29 % of Pb in the kidneys and less than 1 % in other organs. The relationship between the distribution percentage of Cd in the studied organs can be presented as: liver > kidneys > brain > lungs > heart > spleen. The relationship between the distribution percentage of Pb can be presented as: liver > kidneys > brain > spleen > heart > lungs. Significantly (*P* < 0.05), more Cd and Pb were accumulated in total in the organs of rats receiving the metals in drinking water.

## Introduction

Toxic metals present in air, water, and soil have all caused major human health problems in various parts of the world [1]. Humans are exposed to toxic metals by consuming contaminated food and drink. The toxicity of cadmium (Cd) and lead (Pb) can be a cause of miscarriages and developmental disorders. They have a toxic effect on the central nervous system. In addition, they are mutagenic, teratogenic, carcinogenic and embryotoxic [2, 3]. Cd and Pb very easily penetrate through cell membranes in the organism and demonstrate an ability to accumulate in tissues [4, 5]. In soft tissues, the half-life of Cd is between 5 and 30 years [4], while for Pb about 30 days [6]. The distribution of Cd and Pb in the organs is uneven [7]. The degree to which metals are absorbed and accumulated in soft tissues is determined both by factors directly linked with the animal (e.g. age, gender, fitness, physiological condition) and factors not linked with the animal (e.g. the dose, chemical form) [8–10]. Studies have shown that young organisms tend to accumulate more metals than adult ones. This is primarily due to the fact that the mucous membranes of young animals have higher permeability and their defence mechanisms are not fully effective [9].

Available literature contains little information about the effect of the source of toxic metals (drinking water or feed) administered orally on the degree of their accumulation in the organs of young animals, despite abundant information existing on the extent to which Cd and Pb accumulate in the tissues of various species of animals [11, 12]. Also, no data is available comparing and analysing the degree of accumulation of Cd and Pb in the organs during oral exposure to these metals consumed with water or feed. It is essential to explore this topic since the toxic effect of metals on the organisms of animals and humans is directly determined by their concentration in the organism, which is due to the fact that both metals display similar effects which can be cumulative. The objective of the study was to verify to what extent Cd and Pb accumulate in the organs of growing rats subject to long-term oral exposure in the form of a drinking water or feed.

## Material and Methods

### Experimental Assumptions

The experiment aimed to verify whether the source of toxic metals administered to growing (adolescent) rats orally has an influence on the degree of accumulation of Cd and Pb in the organs (brain, spleen, lungs, heart, liver, kidneys). Since the concentration of Cd and Pb is rarely toxic in natural conditions, in the presented experiment, rats were exposed to doses of these metals not exceeding those occurring in conditions of natural exposure [13–15]. The metals were administered with distilled water or feed, assuming that the source of metals can have an influence on the degree of accumulation of Cd and Pb in tissues. At the same time, it was analysed whether the source of metals has any influence on the distribution of Cd and Pb in the respective organs. Literature does not offer corresponding data. The results obtained can facilitate identification of exact mechanisms of absorption of Cd and Pb in the organism.

### Description of the Experiment and the Animals

The experiment carried out on 36 growing male Wistar rats (aged 35 days, body weight 163.3 ± 11.7 g; purchased from the Medical University of Białystok, Poland, No. 6/2003) was approved by the Ethics Committee at the University of Life Sciences in Lublin. All experiments were carried out in accordance with the criteria outlined in the guiding principles of the European Community Council Directive (89/609/EEC) for the care and use of laboratory animals. Over 12 weeks, the rats were kept separately in plastic cages in an air-conditioned room at invariable conditions (temperature approximately 21 °C, humidity approximately 55 %, day and night cycle 12 h/12 h). The rats had unlimited access to feed and water. They were split into six experimental groups of six rats each, as presented in Table 1: Cd-D, Pb-D, Cd/Pb-D, Cd-F, Pb-F, Cd/Pb-F. The rats received Cd and/or Pb respectively in the amount of 7 mg and/or 50 mg per 1 kg of feed or 1 L of distilled water. Immediately after euthanasia and bloodying of the rats, the following organs were prepared as a whole: brain, spleen, lungs, heart, liver and (both) kidneys. The organs were flushed with ice-cold physiological saline (0.9 % NaCl) and dried at room temperature for 5 min. Afterwards, they were weighed as a whole, placed in plastic bags and frozen at −20 °C for 2 weeks until chemical analysis.

**Table 1:** Experimental design

Experimental groups	Cd-D	Cd-F	Cd/Pb-D	Cd/Pb-F	Pb-D	Pb-F
Treatment^a^	7 mg Cd^b^	7 mg Cd^b^	7 mg Cd^b^ + 50 mg Pb^c^	7 mg Cd^b^ + 50 mg Pb^c^	50 mg Pb^c^	50 mg Pb^c^
Source of metals	Drinking water	Feed	Drinking water	Feed	Drinking water	Feed^d^
Access to feedd	Free	Free	Free	Free	Free	Free
Access to drinking waterd	Free	Free	Free	Free	Free	Free
Number of rats	6	6	6	6	6	6
Duration of exposure	12 weeks	12 weeks	12 weeks	12 weeks	12 weeks	12 weeks
Euthanasia^e^	At 12 week	At 12 week	At 12 week	At 12 week	At 12 week	At 12 week

^a^Cd and Pb were supplied at doses not exceeding values equivalent to exposure of humans in exposure conditions, presented by [10–12]

^b^CdCl_2_ (cadmium chloride)

^c^(CH_3_COO)_2_Pb (lead acetate)

^d^feed and drinking water intake were measured daily

^e^Starved for 24 h, afterwards put down in CO_2_ and having the spinal cord broken

### Preparation of Experimental Solutions and Experimental Feed

Distilled water for experimental solutions was prepared each time in our own laboratory. Twice a week it was checked for contamination. CdCl_2_ (POCH S.A., Lublin, Poland) and/or (CH_3_COO)_2_Pb (POCH S.A., Lublin, Poland), weighed with an accuracy of 3 decimal places, was dissolved in the water to obtain concentrated solutions containing 700 mg Cd/L and/or 5000 mg Pb/L. The resulting solutions were stored at room temperature in glass bottles tightly plugged with a cork. The concentrated solutions were used each time to prepare experimental drinking water. Experimental feed was prepared in our own laboratory: standard feed for laboratory animals was ground by mechanical methods and mixed with water-based solutions of CdCl_2_ and/or (CH_3_COO)_2_Pb to obtain feed containing 7 mg of Cd and/or 50 mg of Pb per 1 kg [16]. The feed was mechanically mixed and granulated.

### Chemical Analyses

The samples of tissues (~3 g) were homogenised and dry mineralised at 450 °C for 12 h [16]. The content of Cd and Pb in the ashed samples was determined by the GF AAS method (SpectrAA 880, Varian, USA; atomisation in a graphite furnace, Zeeman background correction). Determination parameters were as follows: Cd λ = 228.8 nm, LOD 0.001 mg/kg, LOQ 0.004 mg/kg; Pb λ = 217.0 nm, LOD 0.011 mg/kg, LOQ 0.03 mg/kg. Three parallel determinations were performed for each sample. The differences in results were on average 5.5 % for Cd and 4.6 % for Pb. Precision was checked against reference material CRM-185 (Institute for Reference Materials and Measurements in Geel, Belgium) and a blank sample. The recovery rate of Cd and Pb averaged 95 %. Detailed methods used to prepare samples for analyses and the parameters for determination of Cd and Pb are presented in another work [16].

### Calculation and Statistical Analysis

The average weekly intake of Cd and Pb with water or feed per 1 kg of body weight was calculated for each of the groups according to the following rule: total metal intake for 12 weeks × 100/average BW after 12 weeks of exposure. The content of Cd and Pb in tissues was calculated per overall weight of the organ. STATISTICA 6.0 software was used to calculate the arithmetic means. Differences between average values obtained in groups in which the source of Cd and Pb was different were evaluated by means of a one-factor variance analysis ANOVA using the *t*-Student-Newman-Keuls test. *P* < 0.05 was assumed to be a statistically significant level. The degree of accumulation of Cd and Pb in the organs was calculated from the following formula: the content of Cd or Pb in organs × 100 / average intake of Cd or Pb in the form of drinking water or feed. The distribution (%) of Cd and Pb in the studied organs was calculated according to the following rule: content of Cd or Pb in respective organs × 100 / total content of Cd or Pb in all organs.

## Results

### The Accumulation of Cd and Pb

The levels of Cd and Pb calculated per overall weight of the organs are shown in Table 2; the highest content was found in the liver (36.2–44.3 μg Cd and 431.4–508.2 μg Pb) and kidneys (16.3–23.7 μg Cd and 170.4–182.6 μg Pb). In the brain, spleen, lungs and heart, the level of these metals did not exceed 0.32 μg Cd and 3.5 μg Pb.

**Table 2:** Accumulation of Cd and Pb in total organs after 12 weeks of exposure

Groups	Brain	Spleen	Lungs	Heart	Liver	Kidneys
	Cd, μg					
Cd-D	0.212 ± 0.03	0.037 ± 0.001	0.143 ± 0.013	0.042 ± 0.003	36.25 ± 1.22	16.32 ± 0.48
Cd-F	0.260 ± 0.01	0.034 ± 0.001	0.075 ± 0.005	0.041 ± 0.004	44.27 ± 3.29	23.75 ± 2.13
Cd/Pb-D	0.299 ± 0.04	0.053 ± 0.004	0.226 ± 0.031	0.033 ± 0.002	44.24 ± 4.11	21.59 ± 1.25
Cd/Pb-F	0.318 ± 0.03	0.070 ± 0.005	0.152 ± 0.008	0.057 ± 0.004	39.76 ± 2.04	23.18 ± 1.38
	Pb, μg					
Pb-D	3.280 ± 0.11	1.608 ± 0.08	0.901 ± 0.05	0.772 ± 0.04	465.7 ± 21.6	170.4 ± 9.25
Pb-F	2.415 ± 0.09	2.154 ± 0.12	0.783 ± 0.05	1.175 ± 0.11	464.7 ± 13.1	181.5 ± 10.4
Cd/Pb-D	2.788 ± 0.29	1.603 ± 0.07	1.046 ± 0.08	0.767 ± 0.02	508.2 ± 37.8	182.6 ± 19.2
Cd/Pb-F	2.896 ± 0.13	1.725 ± 0.18	0.616 ± 0.03	1.541 ± 0.08	431.4 ± 19.5	178.1 ± 7.26

### Distribution of Cd

In Cd-D and Cd/Pb-D groups, all the studied organs accumulated in total 0.48–0.5 % Cd from water, while in Cd-F and Cd/Pb-F groups, 0.45–0.47 % Cd from feed (Table 3). The liver accumulated approximately 63 % (Cd/Pb-F group) to more than 68 % (Cd-D group) of Cd taken in by rats over 12 weeks of the experiment, while kidneys approximately 31 % (Cd-D) to approximately 37 % (Cd/Pb-F) of Cd taken in (Table 3). In the Cd-D group, compared to Cd-F group, the accumulation of Cd was significantly higher in the lungs (more than 64 %), spleen and heart (approximately 30 %) as well as in the brain and liver (above 12.5 %) (Table 4). In group Cd/Pb-D, compared to group Cd/Pb-F, the accumulation of Cd was significantly higher in the lungs (nearly 34 %) and liver (12 %), while it was significantly lower in the heart (approximately 90 %), spleen (above 30 %) and in the liver (above 5 %). In total, the organs of rats in Cd-D group accumulated approximately 7.5 % more Cd than in Cd-F group, while the organs of rats in Cd/Pb-D group accumulated approximately 6.5 % more Cd than in Cd/Pb-F group (Table 4).

**Table 3:** Mean consumption of Cd and Pb, cumulative rate (percent of administered dose) of Cd and Pb in total organs after 12 weeks of exposure and distribution of Cd and Pb for 12 weeks of exposure

Groups	Cd intake, mg/kg BW*	Cumulative rate** of Cd, %	Distribution*** of Cd, %						
			Brain	Spleen	Lungs	Heart	Liver	Kidneys	Total
Cd-D	30.24	0.505	0.40 ^C^	0.07 ^A^	0.27 ^B^	0.08 ^A^	68.4 ^E^	30.8 ^D^	100
Cd-F	42.35	0.467‡	0.38 ^C^	0.05 ^A^‡	0.11 ^B^‡	0.06 ^A^‡	64.7 ^E^	34.7 ^D^	100
Cd/Pb-D	40.05	0.484	0.45 ^D^	0.08 ^B^	0.34 ^C^	0.05 ^A^	66.6 ^F^	32.5 ^E^	100
Cd/Pb-F	40.31	0.452‡	0.50 ^D^	0.11 ^B^‡	0.24 ^C^‡	0.09 ^A^‡	62.6 ^F^	36.5 ^E^	100
	Pb intake, mg/kg BW*	Cumulative rate** of Pb, %	Distribution*** of Pb, %						
Pb-D	279.2	0.719	0.51 ^D^	0.25 ^C^	0.14 ^B^	0.12 ^A^	72.4 ^F^	26.5 ^E^	100
Pb-F	310.2	0.634‡	0.37 ^D^‡	0.33 ^C^‡	0.12 ^A^	0.18 ^B^‡	71.2 ^F^	27.8 ^E^	100
Cd/Pb-D	286.1	0.711	0.40 ^D^	0.23 ^C^	0.15 ^B^	0.11 ^A^	72.9 ^F^	26.2 ^E^	100
Cd/Pb-F	274.5	0.644‡	0.47 ^C^‡	0.28 ^B^‡	0.10 ^A^‡	0.25 ^B^‡	70.0 ^E^	28.9 ^D^	100

In the same row, values with different superscript capital letters differ significantly (*P* < 0.05)

*BW* body weight

*Calculated according to the following formula: total metal intake for 12 weeks × 100 / average BW after 12 weeks of exposure

**Cumulative rate was calculated as the ratio of Cd and Pb content in total organs to the consumption of Cd and Pb after 12 weeks of exposure

***The total content of Cd or Pb in the organs was assumed as 100 %

‡Significant differences compared to drinking water (*P* < 0.05)

**Table 4:** Comparison of the degree of Cd and Pb accumulation in studied organs depending on the source of metals (water or feed)

Groups	Brain	Spleen	Lungs	Heart	Liver	Kidneys	Total
Cd distribution							
Cd-D vs. Cd-F*	+13.2	+30.5	+64.3	+31.0	+12.5	−4.41	+7.46
ANOVA *P*	0.019	0.001	0.000	0.020	0.000	0.120	0.000
Cd/Pb-D vs. Cd/Pb-F *	−2.95	−32.6	+33.8	−90.1	+12.1	−5.05	+6.48
ANOVA *P*	0.857	0.011	0.005	0.031	0.000	0.030	0.019
Pb distribution							
Pb-D vs. Pb-F*	+36.0	−18.8	+24.6	− 40.1	+13.3	+7.65	+11.8
ANOVA *P*	0.004	0.013	0.024	0.033	0.018	0.001	0.001
Cd/Pb-D vs. Cd/Pb-F*	−5.12	−11.6	+36.6	−97.0	+14.1	−2.72	+9.46
ANOVA *P*	0.016	0.009	0.003	0.000	0.017	0.403	0.015

*The values for rats receiving Cd and/or Pb with drinking water (groups Cd-F, Cd + Pb-F and Pb-F) were assumed as 100 %

### Distribution of Pb

In Pb-D and Cd/Pb-D groups, the studied organs accumulated in total approximately 0.71 % of Pb taken in by the rats with water over 12 weeks of the experiment (Table 3). In Pb-F and Cd/Pb-F groups, the organs accumulated in total approximately 0.64 % of Pb taken in with feed. In all groups receiving Pb, the liver showed 70–73 % of Pb accumulated in all the studied organs, and the kidneys accumulated 26–29 % of Pb, while in other organs, the values were not higher than 1 % (Table 3). In Pb-D group, significantly more Pb compared to Pb-F group was accumulated in the brain (36 %), lungs (above 24 %) as well as in the liver and kidneys (13 and 7.6 %, respectively) (Table 4). In Cd/Pb-D group, more (*P* < 0.05) Pb than in Cd/Pb-F group was accumulated in the lungs (nearly 37 %) and liver (14 %) and significantly (*P* < 0.05) less in the heart (97 %), spleen (above 11 %) and brain (above 5 % (Table 3). In total, all the studied organs of rats in Pb-D group accumulated nearly 12 % more Pb (*P* < 0.05) than those of rats in Pb-F group, while in Cd/Pb-D group, the level of Pb was 9 % higher (*P* < 0.05) than in Cd/Pb-F group (Table 4).

## Discussion

Upon oral exposure, the compounds of Pb and Cd are transmitted with blood to respective tissues; about 90 % of Pb is bound by erythrocytes as lead phosphate [17]. Studies have shown that in the organisms of animals subject to oral exposure to Cd and Pb, about 1–8 % of Cd [12] and 10–50 % Pb [18] is absorbed. At the same time, it was recorded that more than 90 % of Pb in this pool is accumulated in bones [6], and the rest in soft tissues. In the presented studies, the rats’ organs (brain, spleen, lungs, heart, liver and kidneys) accumulated in total about 0.5 % Cd and about 0.71 % Pb consumed by rats with water and about 0.46 % Cd and about 0.63 % Pb taken in with feed. Available literature provides no comparative material regarding the accumulation of Cd and Pb in the organs of young rats. Only Ohta et al. [12] demonstrated that the organs of adult rats in total accumulated approximately 0.36–0.54 % Cd consumed in an oral form with drinking water during 5 or 10 weeks of the experiment. Our study involving adult Wistar rats [19] showed values similar to those reported by Ohta et al. [12]; after 12 weeks of exposure, the organs of rats accumulated about 0.3–0.5 % Cd administered in a liquid form and about 0.4 % Cd administered with feed. However, it is known that the organisms of young animals tend to accumulate more toxic metals than those of adults [9]. The presented studies analysed the degree of accumulation of Cd and Pb in the brain, spleen, lungs, heart, liver and kidneys of growing rats and the effect of the method of supply of Cd and Pb (water, feed) on the degree of accumulation of these metals in the studied organs.

### Distribution of Cd and Pb in the Organs

The distribution of Cd and Pb in the organs is not even. It is mostly determined by the rate at which blood flows through the organ, since toxic metals are transported with blood, and by the permeability of cell membranes [5, 7, 20]. In the presented studies, more than 60 % of Cd absorbed by the studied organs accumulated in the liver, more than 30 % in kidneys and less than 1 % in the brain, spleen, lungs and heart. The relationship between the percentage distribution of Cd in the studied organs can be presented as follows: liver > kidneys > brain > lungs > heart > spleen. In the case of Pb: more than 70 % of consumed Pb was accumulated in the liver, about 26–29 % in kidneys and less than 1 % in other organs. The relationship between the distribution percentage can be presented as follows: liver > kidneys > brain > spleen > heart > lungs.

In the presented studies, significantly more Cd and Pb were accumulated in total in the organs of rats receiving this metal with water. The degree of accumulation of Cd and Pb in respective organs was variably dependent on how the metals were administered; the largest differences were recorded in the spleen, lungs and heart. The lungs of rats receiving Cd and Pb with water accumulated nearly twice as much of these metals than rats receiving Cd and Pb in feed. Unexpectedly, the organ that was the least sensitive to this factor was the kidneys. Also, the liver revealed significant, but smaller than in other organs (%), differences in the content of Cd and Pb depending on the method of supply of the metals (feed, drinking water). The results may suggest that regardless of the source of Cd and Pb during oral exposure to low doses of these metals, the liver and the kidneys detoxify the organism equally strongly. It would be interesting to investigate whether these organs respond in the same way when the organism is exposed to different doses of Cd and Pb.

In addition, in the presented studies, differences were recorded between the distribution of Cd and Pb in certain organs when supplied separately (Cd or Pb) and in combination (Cd + Pb – combined exposure). During separate exposure from water compared to separate exposure from feed, more Cd was accumulated in the lungs and Pb in the hearts and brains, and during combined exposure, greater amounts of both Cd and Pb were accumulated in the hearts when the metals were supplied with feed than with water. The results obtained testify that apart from the blood flow rate, the distribution of Cd and Pb in organs is also determined by the source of metals (water, feed) and the type of exposure (separate, combined). The difference between the degree of accumulation of Cd and Pb in the organs during separate and combined exposure can be a result of the antagonism between these metals, which can be a form of defence of the organism against the cumulative effect of Cd and Pb supplied at low doses on a long-term basis. This problem needs to be explained and our team will continue research to investigate this issue.

### Effect of Chelating Agents on the Accumulation of Cd and Pb in the Organs

Cd and Pb are metals whose absorption can be limited by various ingredients found in feed or water. The most important ones are dietary fibre, phytates—in particular inositol hexakisphosphate (IP-6), polyphenols and certain minerals. Dietary fibre is capable of chelating Cd and Pb, while the ability to bind metals depends on the source of origin and fractional composition of dietary fibre and on the presence of carboxyl groups deriving from cellulose, hemicelluloses and pectins as well as phenolic groups contained in lignin [21]. The absorption of Cd and Pb by IP6 and polyphenols is also limited by chelating of these metals [16, 22, 23]. Studies have revealed that when the metals are supplied with feed, the extent to which they are chelated by tannic acid is higher than when they are supplied with water [16]. Interactions between Cd and Pb and other dyads stem from various reasons, but they are mostly limited to displacing Cd and Pb from tissues when a sufficient amount of essential metals is present in the organism. The reduced concentration of Cd in cells is a result of an antagonism or mutual displacement occurring between the ions of zinc, copper, iron, calcium and Cd in cell transport [24–26].

In the presented studies by these authors, Cd and Pb was supplied in distilled water or feed. Metal-chelating components were contained in feed only, which explains why the overall accumulation of the metals was lower in the organs of rats receiving Cd and Pb with feed. In turn, the chemical form of Cd and Pb – CdCl_2_ and (CH3COO)_2_Pb is very easily dissolved in water, which could facilitate much faster transmission of Cd and Pb to the bloodstream of rats receiving the metals with drinking water. Also, they were absorbed faster before the organism was able to fully activate its own defence mechanisms, such as metallothionein, the synthesis of which is induced by metals and which transports them to Paneth cells responsible for metabolizing and detoxifying toxic metals [27]. It can serve as a proof that most toxic metals are absorbed during the first term of exposure. Afterwards, the rate of absorption may decrease. This problem will be investigated in a subsequent experiment. In vitro studies carried out by Chunhabundit et al. [28] demonstrated that the bioactivity of Cd determined by means of the Caco-2 model for Cd was higher when supplied with water than with feed.

## Conclusions

The degree of accumulation and distribution of Cd and Pb in the organs of young animals compared to the amount taken in with water or feed have not been thoroughly investigated yet. The presented studies have shown that this figure could be determined by at least several factors such as the time of exposure, source of metals (water or feed) and type of exposure (separate or combined). It could also be supposed that the studied parameters can be influenced by the form of Cd and Pb and the rate of blood flow through the organ. Further experiments must be carried out to explain why the degree of accumulation of Cd and Pb was different in different organs depending on the method of supply of the metals and why the kidneys and the liver were the most sensitive to this factor. The results obtained may suggest that the source of Cd and Pb have no significant influence on the detoxicating function of these organs. However, it would be interesting to investigate whether these organs respond in the same way when the organism is exposed to different, including very high doses of Cd and Pb. Also, the effect of the time of exposure of rats on the studied parameters must be explored.
